# Cutaneous tuberculosis: An infrequent manifestation of a common pathogen in South Africa

**DOI:** 10.4102/sajid.v38i1.526

**Published:** 2023-06-13

**Authors:** Jennifer K. van Heerden, Alistair G.B. Broadhurst, Ruan S. de Jager, Wesley du Plessis, Nabilah Ebrahim, Ayanda T. Mnguni, Denzil Schietekat, Graeme Meintjes

**Affiliations:** 1 Department of Internal Medicine, Khayelitsha District Hospital, Western Cape Department of Health, Cape Town, South Africa; 2 Wellcome Centre for Infectious Diseases Research in Africa, Institute of Infectious Disease and Molecular Medicine, Faculty of Health Sciences, University of Cape Town, Cape Town, South Africa; 3 Division of General Internal Medicine, Department of Medicine, Faculty of Medicine and Health Sciences, Stellenbosch University and Tygerberg Hospital, Cape Town, South Africa; 4 Division of Dermatology, Department of Medicine, Faculty of Medicine and Health Sciences, Stellenbosch University and Tygerberg Hospital, Cape Town, South Africa; 5 Department of Medicine, Faculty of Health Sciences, University of Cape Town and Groote Schuur Hospital, Cape Town, South Africa

**Keywords:** cutaneous tuberculosis, human immunodeficiency virus, HIV, tuberculosis, TB, South Africa

## Abstract

Cutaneous tuberculosis is an infrequent form of extra-pulmonary tuberculosis, even in high-prevalence settings. We present the case of a patient living with advanced HIV who developed extensive cutaneous tuberculosis. The polymorphic skin lesions were the most striking clinical manifestation of underlying disseminated tuberculosis.

**Contribution:**

This case report highlights an unusual presentation of tuberculosis. Cutaneous tuberculosis has a wide spectrum of clinical presentations and may be under-recognised by clinicians. We recommend early biopsy for microbiological diagnosis.

## Background

Tuberculosis (TB) remains a significant contributor to the global burden of disease and an estimated 10.6 million people fell ill with TB in 2021 (95% uncertainty interval: 9.9–11 million).^1^ Despite the high incidence of TB, often occurring in the setting of human immunodeficiency virus (HIV) co-infection, cutaneous TB is infrequent, comprising less than 2% of all extra-pulmonary forms of TB.^2,3,4^ As an infrequent and possibly under-recognised presentation of TB, there is a paucity of clinical and microbiological data on cutaneous TB in South Africa.

Cutaneous TB predominantly refers to skin disease caused by *Mycobacterium tuberculosis (MTB)*; although, rarely, it can be caused by *Mycobacterium bovis* or develop from the attenuated form of *M. bovis* contained in the *bacillus Calmette-Guerin* (BCG) vaccine.^4^ Additionally, cutaneous hypersensitivity reactions to antigen components of *MTB* are often included as forms of cutaneous TB.^5^ Cutaneous TB may have a wide variety of clinical presentations each with distinct morphologies. This heterogeneity contributes to diagnostic difficulty, delayed diagnoses and ultimately a delay in initiation of effective treatment.

In light of this, we present a patient with an unusual presentation of cutaneous TB and highlight in the discussion the varied presentation of cutaneous TB, to contribute to the literature on this condition in South Africa. We emphasise the need for prompt recognition with early biopsy for microbiological diagnosis and determination of drug sensitivities to guide therapy.

## Case presentation

A 38-year-old woman with advanced HIV disease (antiretroviral treatment experienced with treatment interruption) presented to Khayelitsha District Hospital with multiple cutaneous abscesses and associated constitutional symptoms of loss of appetite, loss of weight and night sweats. She reported a 3-month history of subcutaneous swellings with overlying hyperpigmentation affecting the arms, legs and buttocks, which subsequently formed spontaneous sinuses with drainage of purulent discharge.

The patient had a history of three previous episodes of drug-sensitive TB and had completed anti-TB treatment on all occasions (Table 1). She had recently been admitted to the surgical department where she was diagnosed with lower limb cellulitis complicated by soft tissue collections. During that admission, an incision and drainage procedure was performed, and the patient received an oral course of amoxicillin and clavulanic acid. Routine microscopy, culture and sensitivity of the pus did not identify any bacterial pathogen; however, a specific mycobacterial culture was not requested.

**TABLE 1:** Previous episodes of tuberculosis.

Year	Type (site) of tuberculosis	Basis of diagnosis
2017	Disseminated tuberculosis	Diagnosed at explorative laparotomy. Mycobacterial culture on intra-abdominal tissue and aspirate samples: *MTB* complex, sensitive to rifampicin and isoniazid
2020	Pulmonary tuberculosis	*Xpert*^®^ *MTB/Rif Ultra* on sputum: *MTB* complex detected, sensitive to rifampicin
2021	Disseminated tuberculosis	Urinary LAM positive Mycobacterial culture on blood: *MTB* complex, sensitive to rifampicin and isoniazid

LAM, lipoarabinomannan; *MTB, Mycobacterium tuberculosis*.

**FIGURE 1:**
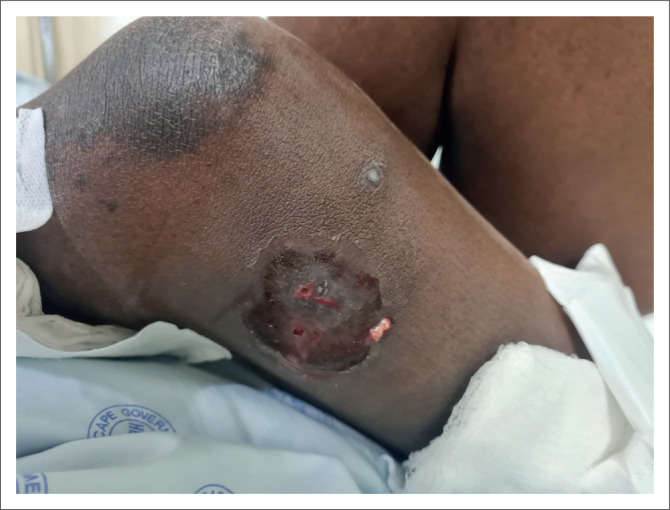
Lower limb subcutaneous collection with draining sinuses.

**FIGURE 2:**
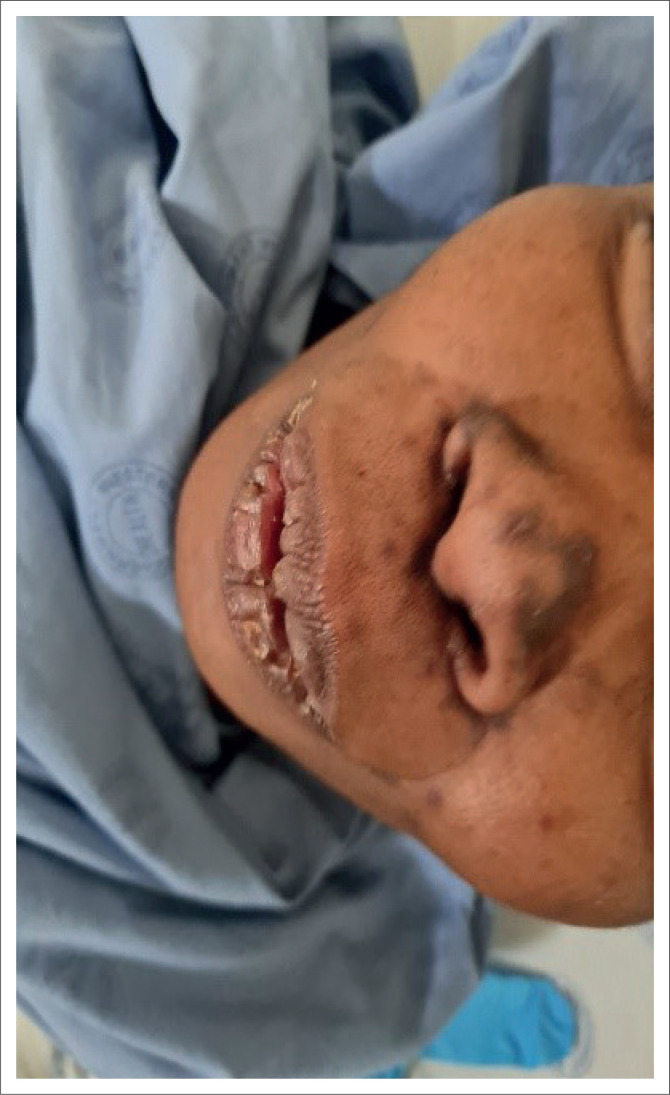
Hyperpigmented papules and plaques affecting the perioral and nasal area.

On admission to the internal medicine department, the patient had a tachycardia and a documented fever. She was cachectic with pallor and generalised lymphadenopathy. She had multiple skin lesions with a widespread distribution, affecting predominantly the lower limbs, perianal area, hands and face. The lower limb and perianal lesions involved subcutaneous collections with overlying hyperpigmented patches and central fluctuance. Certain of these collections had ulcerated with draining sinuses (Figure 1). Her facial lesions were hyperpigmented papules and plaques predominantly affecting the nasal area and associated with crusting suggestive of lupus vulgaris (Figure 2). Initial blood results showed acute kidney impairment, normocytic anaemia and an elevated C-reactive protein (Table 2).

**TABLE 2:** Laboratory investigations on or after the index admission.

Investigation	Value†	Peak	Nadir	Reference range‡
Sodium (mmol/L)	126	134	126	136–145
Potassium (mmol/L)	4.2	5.8	2.9	3.5–5.1
Urea (mmol/L)	10.4	17.6	5.1	2.1–7.1
Creatinine (μmol/L)	135	151	83	98–107
Haemoglobin (g/dL)	8.6	9.3	5.9	12.0–15.0
Mean corpuscular volume (fL)	81.4	100.0	80.9	78.9–98.5
White cell count (×10^9^/L)	7.02	7.02	3.38	3.90–12.60
Platelet count (×10^9^/L)	196	196	40	186–454
Alanine transaminase (U/L)	52	89	52	7–35
Total bilirubin (μmol/L)	87	104	30	5–21
Albumin (g/L)	13	14	11	35–52
C-reactive protein (mg/L)	78	207	58	< 10
Ferritin (μg/L)	1767	1767	-	11–307
International normalised ratio	1.42	1.42	0.94	0.8–1.2
D-Dimer (mg/L)	7.46	7.46	4.59	0.0–0.25
Fibrinogen (g/L)	1.4	2.0	1.4	2.0–4.0
Bacterial culture on blood	No growth	-	-	-
CD4 lymphocyte count (cells/μL)	115	-	-	332–1642
HIV viral load (copies/mL)	> 10 000 000§	-	-	-
Serum cryptococcal antigen	Negative	-	-	-
Rapid plasma reagin	Non-reactive	-	-	-
**Lumbar puncture**				
Protein (g/L)	1.24	-	-	0.15–0.45
Glucose (mmol/L)	2.3	-	-	-
Polymorphonuclear cells (cells/μL)	0	-	-	-
Lymphocytes (cells/μL)	8	-	-	-
Erythrocytes (cells/μL)	1	-	-	-
Xpert^®^ MTB/Rif Ultra	*MTB* not detected	-	-	-
Cryptococcal antigen	Negative	-	-	-
MC&S	No bacteria observed, no growth after 5 days	-	-	-

CD4, cluster of differentiation 4; HIV, human immunodeficiency virus; MC&S, microscopy, culture and sensitivity; MTB, *Mycobacterium tuberculosis*.

† , Values displayed are those from samples taken on admission to hospital or as the first available value after admission;

‡ , Reference range values quoted are taken from the National Health Laboratory Service (NHLS) reference ranges;

§ , Equates to > 7.00 log_10_ (copies/mL).

## Differential diagnosis

The differential diagnosis for this patient’s initial presentation was relatively broad, with infectious causes being the most important considerations.

Firstly, *Staphylococcus aureus* (including methicillin-resistant *S. aureus*) and *Streptococcus pyogenes* are the two most significant bacterial pathogens causing cutaneous abscesses.^6^ The negative bacterial cultures from the surgical drainage procedure and the chronic course of this presentation made us consider these causes unlikely. Secondly, although far less common, actinomycosis should be considered in this patient with significantly compromised immunity. This sub-acute or chronic suppurative, granulomatous infection caused by *Actinomyces* spp. may cause purulent, mass-like lesions with draining sinuses, similar to those described here.^7^

Mycobacterial opportunistic diseases such as *MTB* and nontuberculous mycobacteria are important aetiologies to consider, as well as other acid-fast bacilli, such as *Nocardia* spp., that may mimic TB presentations.^8^ Cutaneous syphilitic gumma, as a manifestation of tertiary syphilis, may also be considered.^4^

Deep fungal infections may have chronic cutaneous manifestations similar to this case.^9,10^ The subcutaneous mycoses such as sporotrichosis and mycetoma, as well as the systemic mycoses such as blastomycosis, cryptococcosis and histoplasmosis were all considered in this patient with profound immune compromise.^4,9^

## Case management

### Investigations

Laboratory findings are listed in Table 2. Blood cultures showed no growth after 5 days and serum cryptococcal antigen and rapid plasma reagin were non-reactive. The patient developed delirium on day three of admission, necessitating a lumbar puncture for cerebrospinal fluid (CSF) analysis that revealed a lymphocytic pleocytosis and an elevated protein that was suggestive of TB meningitis despite a negative CSF GeneXpert.

A pus aspirate from the largest skin lesion was performed, from which acid-fast bacilli were observed on microscopy and *MTB complex* was detected by real-time polymerase chain reaction (RT-PCR) testing, with the *MTB* determined as sensitive to rifampicin (Xpert^®^ MTB/Rif Ultra, Becton Dickinson, United States). Prompt consultation with dermatology was sought for a skin biopsy, where *MTB* was detected by Xpert^®^ MTB/Rif Ultra on the tissue sample and histopathology showed organising inflammation (Table 3).

**TABLE 3:** Microbiological laboratory investigations.

Investigation	Result
**Urinary LAM**	Positive
**Pus aspirate from skin lesion on lower limb**	-
Xpert^®^ MTB/Rif Ultra	*MTB* complex detected, sensitive to rifampicin
TB MC&S	AFB observed *MTB* complex isolated, sensitive to rifampicin and isoniazid
Bacterial MC&S	Methicillin Sensitive *Staphylococcus aureus* isolated
**Gastric aspirate**	-
Xpert^®^ MTB/Rif Ultra	*MTB* complex detected, sensitive to rifampicin
TB MC&S	AFB observed *MTB* complex isolated
**Blood mycobacterial culture**	Negative
**Skin biopsy**	
Xpert^®^ MTB/Rif Ultra on tissue homogenate	*MTB* complex detected, sensitive to rifampicin
Bacterial and fungal MC&S	No bacterial or fungal growth
Histopathology	Organising inflammation and fibrosis
	Fibrosis, telangiectasia and numerous plasma cells and lymphocytes throughout the reticular dermis extending into the subcutaneous fat.
	Abundant hemosiderin pigment and red blood cell extravasation are present.
	Macrophages are prominent in areas but granulomatous inflammation is absent.

AFB, acid fast bacilli; LAM, lipoarabinomannan; MC&S, microscopy, culture and sensitivity; MTB, *Mycobacterium tuberculosis*; TB, tuberculosis.

Concurrently to the investigation of the skin lesions, laboratory evidence for disseminated TB was sought. A urinary lipoarabinomannan test was positive and a gastric aspirate demonstrated *MTB* complex, detected by Xpert^®^ MTB/Rif Ultra, with *MTB* complex isolated by mycobacterial culture. Radiological investigations provided further evidence supporting a diagnosis of disseminated TB with upper lobe cavitation seen on chest radiography and multiple hypoechoic splenic lesions detected by abdominal ultrasound (Table 4).

**TABLE 4:** Radiological investigations.

Investigation	Result
Chest radiograph	Upper lobe cavitation.
Abdominal ultrasound	Enlarged spleen containing hypoechoic lesions. Liver noted as enlarged and echogenic.
Computed tomography of the brain	No intracranial space-occupying lesions or abnormal contrast enhancement.

### Outcome and follow-up

The patient was started on anti-TB therapy with rifampicin, isoniazid, ethambutol and pyrazinamide. Corticosteroids were added to the treatment regimen after TB meningitis was diagnosed. The patient was transferred to a designated TB hospital where she continued anti-TB therapy and antiretroviral treatment was subsequently initiated. She had a good clinical response to treatment with marked improvement in all skin lesions and no neurological impairment on discharge from this facility. The discharge plan was to complete a total duration of 9 months of anti-TB therapy on an outpatient basis.

## Discussion

This case report demonstrates that multiple cutaneous abscesses in an immunosuppressed individual should alert the treating clinician to consider cutaneous TB. However, this is only one possible form of cutaneous TB as there are many varied presentations of the condition, depending on multiple factors such as the route of transmission, the host’s cellular immunity, the proximity to lymph nodes and the microbial virulence.^11^

The spectrum of cutaneous TB ranges from inflammatory papules and verrucous plaques to chronic ulcerative lesions and cold abscesses.^3,4,11^ Cutaneous TB can be broadly categorised according to the mechanism of infection (outlined in Table 5) as this determines the type of lesion.^12^ For example, spread from an exogenous source (direct inoculation of bacilli) may cause a tuberculous chancre, whereas spread from an endogenous source may cause other manifestations of cutaneous TB, such as lupus vulgaris. Although the mode of infection may not be clear in all cases, in our patient the spread was almost certainly endogenous via haematogenous dissemination.^4,11^

**TABLE 5:** Classification of cutaneous tuberculosis.

Category	Examples
Exogenous cutaneous tuberculosis	Tuberculous chancre† Tuberculosis verrucosa cutis
Endogenous cutaneous tuberculosis	By contiguity or autoinoculation: scrofuloderma†, tuberculosis orificialis†, lupus vulgaris By haematogenous dissemination: lupus vulgaris, tuberculous gumma† and acute miliary tuberculosis†
Tuberculids	Papulonecrotic tuberculid Lichen scrofulosorum
Cutaneous tuberculosis secondary to BCG vaccination	BCG cutaneous abscess

*Source:* Dos Santos JB, Figueiredo AR, Ferraz CE, Oliveira MH, Silva PG, Medeiros VL. Cutaneous tuberculosis: Epidemiologic, etiopathogenic and clinical aspects - part I. An Bras Dermatol. 2014;89(2):219–228. https://doi.org/10.1590/abd1806-4841.20142334

BCG, *Bacillus Calmette-Guerin*.

† , Indicates multibacillary forms of cutaneous tuberculosis.

In another classification analogous to the Ridley and Jopling classification for leprosy, the bacterial load can be used to classify cutaneous TB. Multibacillary forms include scrofuloderma, acute miliary tuberculosis and tuberculous gumma, whereas paucibacillary forms include verrucous tuberculosis and tuberculids.^4,5^ This classification also provides insight into the immune pathogenesis of cutaneous TB and its relationship to the patient’s cellular immunity to *MTB*. For example, tuberculids, a paucibacillary form of cutaneous TB, develop as immunological reactions to antigenic components of mycobacteria and usually occur in patients with a good cellular immunity.^13^ Conversely, in our patient who was severely immunocompromised, the lesions were multibacillary, as evidenced by the positive stain for acid-fast bacilli on the pus aspirated.

The multiple subcutaneous tuberculous abscesses in our patient are analogous to ‘tuberculous gumma’, lesions that characteristically affect individuals during periods of decreased cellular immunity, such as advanced HIV.^11^ Tuberculous gumma typically affects the trunk and lower extremities and may ulcerate and drain caseous material or pus.^11^ Another manifestation of cutaneous TB with a similar mechanism of spread and affecting a similar patient population is acute miliary cutaneous TB, where lesions appear as generalised papules, vesicles or pustules.^12,14^ These two manifestations of cutaneous TB are important to recognise as they may be associated with poor outcomes if therapy is delayed given underlying disseminated TB.

The pattern of cutaneous facial involvement observed in our case was suggestive of lupus vulgaris; however, other forms of cutaneous TB may present similarly and were considered as possible alternative diagnoses. For example, TB cutis orificialis may cause ulcerative cutaneous and mucosal lesions in periorificial areas and usually occurs in individuals with TB at other sites as well as those with impaired immunity.^3,4^ Additionally, non-mycobacterial cutaneous manifestations of systemic diseases such as syphilis and systemic mycoses formed an important part of the differential diagnosis of the facial lesions in this case.^3,4,5^

It is imperative to obtain early tissue samples in patients with suspected cutaneous TB. Culture remains the gold standard for making a microbiological diagnosis of TB; however, nucleic acid amplification testing (such as by the Xpert^®^ MTB/Rif Ultra test) has been shown to be a sensitive and specific diagnostic test on pus aspirates as well as tissue homogenate.^2,15,16^ Xpert^®^ MTB/Rif Ultra performed on both the pus aspirate and tissue were positive for *MTB* in our patient. This test has the benefit of rapid turnaround time with rifampicin sensitivity testing, allowing for early treatment. Histopathology of cutaneous TB shows inflammation and, classically, caseating granulomas.^4^ The absence of caseating granulomas in our patient is unusual; however, it is reported in approximately 10% of cases of cutaneous TB and likely related to profound immunosuppression in our patient.^17^

In our patient, the subcutaneous abscesses present during the previous admission that did not resolve with incision, drainage and antibiotics were almost certainly a missed manifestation of cutaneous TB as no TB tests were requested at the time. This phenomenon is described in other case reports, where patients with cutaneous TB often receive multiple courses of antibiotics prior to diagnosis.^2^ Although *S. aureus* was cultured on pus aspirate in our case, it was not considered causative but rather a secondary infection that entered via a draining sinus.

Treatment of cutaneous TB does not differ from other forms of TB and our patient showed a good response to effective combination therapy. Concurrent TB at other sites should be fully investigated as this may guide the duration of treatment and the need for adjuvant glucocorticoids.
